# Effect of the suture technique on postoperative pain, swelling and trismus after removal of lower third molars: A randomized clinical trial

**DOI:** 10.4317/medoral.20307

**Published:** 2015-02-07

**Authors:** Cosme Gay-Escoda, Laila Gómez-Santos, Alba Sánchez-Torres, José-María Herráez-Vilas

**Affiliations:** 1 MD, DDS, MS, PhD. Chairman and Professor of Oral and Maxillofacial Surgery, School of Dentistry, University of Barcelona. Director of Master’s Degree Program in Oral Surgery and Implantology (EHFRE International University/UCAM/FUCSO).Coordinator/Researcher of the IDIBELL Institute. Head of Oral Surgery, Implantology and Maxillofacial Surgery Department of the Teknon Medical Center, Barcelona (Spain); 2 DDS. Fellow of the Postgraduate Programme in Oral Surgery and Implantology. School of Dentistry of the University of Barcelona (Spain); 3 DDS. Fellow of the Master of Oral Surgery and Implantology. School of Dentistry of the University of Barcelona (Spain); 4 DDS, MS. Professor of the Master of Oral Surgery and Implantology. School of Dentistry of the University of Barcelona. Researcher of the IDIBELL Institute. Barcelona (Spain)

## Abstract

**Background:**

To evaluate the intensity of pain, swelling and trismus after the removal of impacted lower third molars comparing two different suture techniques of the triangular flap: the complete suture of the distal incision and relieving incision and the partial suture with only one suture knot for closure of the corner of the flap and the closure of the distal incision, without suturing the relieving incision.

**Material and Methods:**

A prospective, randomized, crossover clinical trial was conducted in 40 patients aged from 18 to 45 years who underwent surgical extraction of impacted lower third molars at the Department of Oral Surgery in the Odontological Hospital of the University of Barcelona during the year 2011. Patients were randomly divided in 2 groups. Two different techniques (hermetical closure and partial closure of the wound) were performed separated by a one month washout period in each patient. Postoperative pain, swelling and trismus were evaluated prior to the surgical procedure and also at 2 and 7 days post operatively.

**Results:**

No statistically significant differences were observed for pain (p<0.06), trismus (p<0.71) and swelling (p<0.05) between the test and the control group. However, the values of the three parameters related to the test group were lower than those for the control group.

**Conclusions:**

Partial closure of the flap without suturing the relieving incision after surgical extraction of lower third molars reduces operating time and it does not produce any postoperative complications compared with complete closure of the wound.

**Key words:** Third molar, surgical flaps, suture techniques, postoperative pain, swelling, trismus.

## Introduction

Removal of impacted lower third molars is one of the most common surgical procedures in Oral Surgery (1-14). Pain, swelling and trismus are considered as immediate postoperative tissue reactions following third molar surgery and they have been commonly related with the length of the surgical intervention, the surgical difficulty and operative trauma (2-9,12-16). In some cases, complications can occur, which are unwanted reactions that may not necessarily follow the surgical procedure, including: bleeding or hemorrhage (4,5,8,15), postoperative infections like dry socket (3-5,8,15), nerve injury, delayed healing and the creation of a periodontal pocket in the distal aspect of the adjacent second molar (5,6).

Primary and secondary closure are used for the wound management after extraction of impacted lower third molars. There have been many studies to determine the effect of these wound closure techniques on postoperative pain, swelling and trismus.

Some of them compare these variables by means of using different suture techniques (3,5,7,9,10,12,14,15), different type of flaps (6,8,11) and even with the use of tube drains (2,13,17-20). The aim of the present study is to compare postoperative pain, swelling and trismus between primary or complete closure of the wound with secondary or partial closure, which consists in suturing hermetically the distal incision of the triangular mucoperiosteal flap and using one suture knot at the corner of the triangular flap, without suturing the relieving incision completely.

The null hypothesis is that there is no a reduction of pain, swelling and trismus after the third molar extraction with the use of complete closure of the flap against partial closure.

The alternative hypothesis is that after the third molar extraction, partial closure of the wound reduces pain, swelling and trismus compared to the complete closure.

## Patient and Methods

A prospective, randomized, crossover clinical trial was conducted. The study protocol was approved by the Ethics Committee from the Odontological Hospital of the University of Barcelona. The surgical procedure was performed during the year 2011 by a second-year resident and variables were taken by the same fellow of the Master of Oral Surgery and Implantology at the University of Barcelona (Spain).

The sample size was calculated using the statistical program G* Power 3.0. (Heinrich-Heine-Universität, Düsseldorf, Germany), with an alpha value of 0.05, a statistical power of 95%. In order to detect differences of 5 mm in the variable trismus (mm) at 48 hours we used 35 and 40 mm for group A and B, respectively, with a standard deviation of 8 mm and assumed a dropout rate of 10%.

The initial sample size was 57 patients from which 17 were excluded because of missing information in their medical files. Therefore the final sample size consisted of 40 patients. Randomization blocks were created in order to randomly divide patients in 2 groups. A randomization table was used to select the third molar for extraction and to determine whether the patients were included in group A or group B. Patients belonging to group A (n=20) underwent surgical extraction of 4.8 or 3.8 with complete closure of the flap (hermetic closure of the distal incision and the relieving incision), according to the randomization table. After a one month washout period, the surgical extractions for the contra lateral molars with partial closure of the flap (hermetic suturing of the distal incision and just one-knot at the vestibular corner of the flap) were performed. Patients belonging to group B (n=20) followed the surgical procedures conversely to group A.

Inclusion criteria were: 1) patients with an indication for extraction of both lower third molars with a symmetrical grade of impaction assessed using the Pell and Gregory classification; 2) healthy patients (ASA I) or patients with systemic mild disease with no functional limitations (ASA II) and with no objective contraindication for surgical procedure; 3) age range: 18-45 years; 4) patient willing to participate in the study that completes follow-up visits and signed the informed consent for treatment. Exclusion criteria were: 1) patients with systemic diseases ASA III, ASA IV and ASA V; 2) patients using antibiotic pre medication or using medication that would affect wound healing; 3) patient with acute pericoronaritis or severe periodontal disease; 4) patients allergic to the drugs or local anesthesia used in the study; 5) patients undergoing more than one extraction during the same surgical procedure.

- Data collection

The variables assessed were both clinical and radiographic. Clinical variables were age, gender, smoking habit, history of pericoronaritis, maximum interincisal distance (measured with vernier callipers), severity of pain (using a visual analogue scale from 0 to 10) and 3 facial measurements (horizontal, oblique and vertical) in order to determine facial swelling, using measuring tape. The horizontal measure is the distance from the corner of the mouth to the attachment of the ear lobe following the bulge of the cheek, the vertical measure is the distance from the outer can thus of the eye to the angle of the mandible and the oblique one is the distance from the corner of the mouth to the angle of the mandible. Radiologic variables were taken from a previous orthopantomography (with less than 6 months) and the level of impaction was assessed using the Pell and Gregory classification. Post surgical variables were also registered: 1) duration of surgical procedure since incision until last knot tying; 2) ostectomy and/or coronal and/or root sectioning; 3) periosteal integrity.

- Surgical protocol

**Figure 1:**
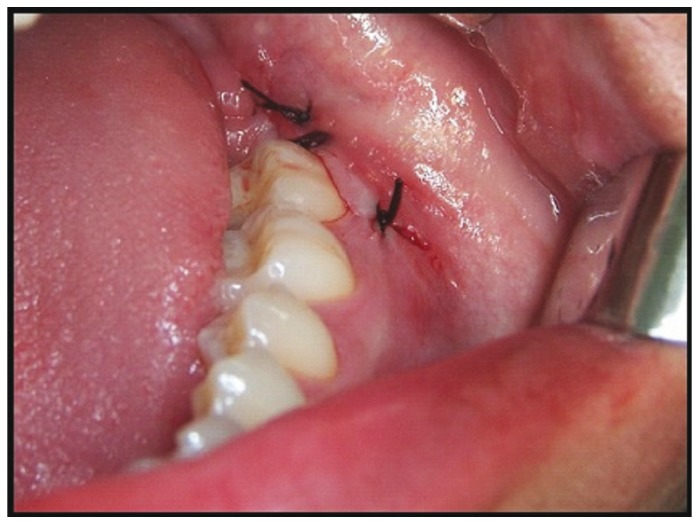
Surgical technique used in the test group for the hermetic suture of the distal incision and the placement of one suture knot in the corner of the flap, without suturing de relieving incision.

Surgical extraction was done under local anesthesia, using a 4% articaine (1:100.000 epinephrine) anesthetic solution (Artinibsa®, Inibsa, Barcelona, Spain). A crestal incision with a relieving incision at mesial part of the adjacent second molar that crossed the mucogingival line, with a length equal or greater than 10 mm, was performed. The mucoperiosteal flap was raised and ostectomy was performed using low-speed hand pieces (maximum 40.000 rpm) and a number 8 tungsten carbide bur. Curettage and irrigation of the surgical bed was performed using sterile distilled water (Braun medical, Barcelona, Spain). Sutures were done with 3-0 silk with a C16 needle (Aragó, Barcelona, Spain). The suture technique in test group, as shown in figure 1, consisted in one suture knot tied at the corner of the triangular flap and hermetic suture at the distal aspect of the adjacent second molar. On the contrary, a hermetic suture of distal and relieving incisions of the triangular flap was made in control group. Finally, patient was instructed to bite on sterile gauze for 30 minutes.

All patients were given written information regarding to postoperative instructions and medication: Amoxicillin EFG (Normon, Madrid, Spain) 750 mg/tablet, 1/8 hours during 4 days; Ibuprofen EFG (Normon, Madrid, Spain) 600 mg/tablet, 1/8 hours during 2 days and if needed, until the third day; and Chlorhexidine 0.12% mouth wash rinse (Lacer, Cerdanyola, Spain), 15 mL/12 hours during 15 days, starting 24 hours after surgery. The rescue analgesic was Metamizol EFG (Normon, Madrid, Spain) 575mg/tablet, 2/8 hours. Registered variables were collected prior to surgical procedure, and at 2 and 7 days during the follow-up period.

All patients underwent postoperative follow-up visits at second and seventh days after surgery and the following variables were registered: facial measurements, mouth opening and the severity of pain. The presence or absence of postoperative complications was also measured in terms of dehiscence of the wound, bleeding and infections as well as the number of anti-inflammatory tablets and rescue analgesics taken by the patient.

- Statistical method

All data obtained were introduced in a database and processed with the SPSS version 15.0 statistical package (SPSS, Chicago, USA). Analysis of Variance (ANOVA) was used for repeated measures, as well as Chi-square and Pearson tests. *P* value < 0.05 was considered statistically significant.

## Results

The final sample size consisted of 40 patients that followed the surgical extraction of both lower third molars during the year 2011. Nineteen were males (47.5%) and 21 were females (52.5%). Age interval ranged from 18 to 44 years with an average age of 25.2 years.

**Figure 2:**
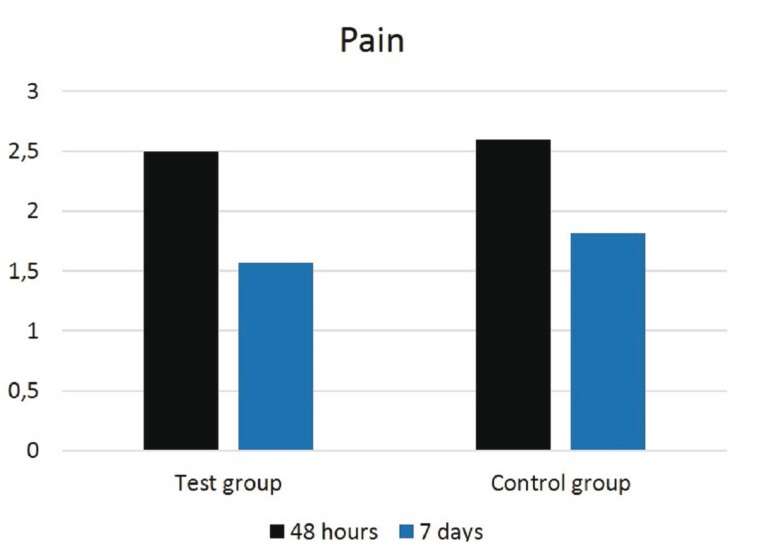
Graph showing pain intensity at 48 hours and at 7 days after surgery in the test and control groups.

**Figure 3:**
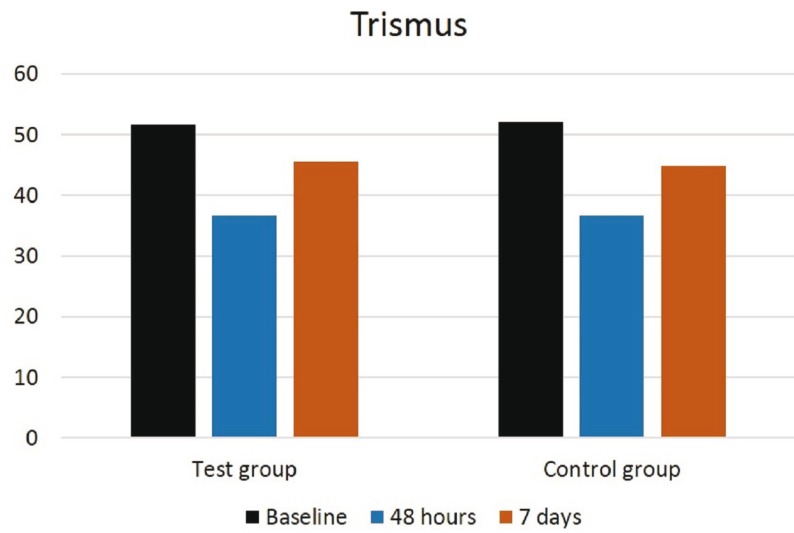
Graph showing mouth opening prior to surgery, at 48 hours and at 7 days after surgery in the test and control groups.

**Figure 4:**
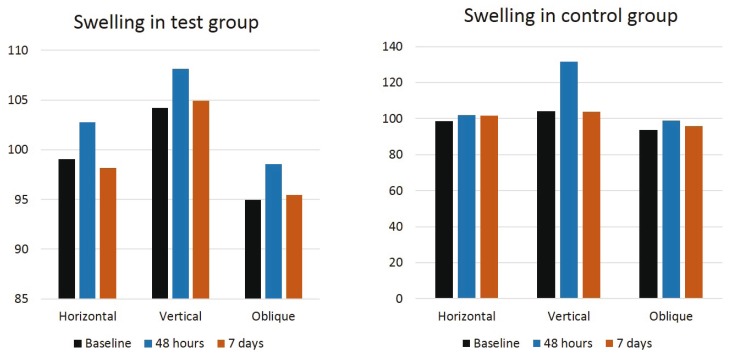
Graphics showing the extent of swelling prior to surgery, at 48 hours and at 7 days after the surgical extraction in the test and control groups.

No statistically significant differences were found to be related to pain (*p*<0.06) at 48 hours and 7 days between the different suture techniques in group A and B, as shown in figure 2, although pain scores were greater in the complete closure than in the partial closure. There were no significant differences for trismus between none of them by measuring the mouth opening (*p*<0.71) before surgery, at 48 hours and at 7 days after surgery, as seen in figure 3. Regarding swelling, there were no significant differences for horizontal (*p*<0.73), vertical (*p*<0.37) and oblique (*p*<0.83) facial measurements taken prior, at 48 hours and at 7 days after surgery, between the distinct suture techniques in both groups. However, as shown in figure 4, greater values for facial vertical distance were reported for the complete closure in both groups.

## Discussion

Swelling, trismus and pain are the most important indicators following surgical extraction of impacted lower third molars (2-9,10,12-15,18).

The level of swelling was determined by means of horizontal, vertical and oblique facial measurements. In the literature, the use of visual (7,12,15) and even verbal (8) analogue scales, the use of an extra oral cephalostat (2,7), photographic techniques (2,7,12,15), computed tomography and stereo photographic techniques (7,12,15) has also been described.

The intensity of pain was evaluated using a visual analogue scale, which is considered to be an effective tool to assess subjective clinical parameters (7,12,15).

In this study, there are not statistically significant differences for trismus, pain and swelling, comparing both type of sutures. However, these variables are lower for the partial closure technique.

A study similar to ours conducted by Osunde *et al*. (14) evaluated the role of the suture technique in relation to postoperative complications and concluded that there were no significant differences between the complete closure and a one-knot in the corner of the flap, although the group with partial closure presented a reduction in postoperative variables (pain, swelling and trismus). Likewise, Maria *et al*. (12) found a lower level of postoperative variables in the group with a secondary closure, as well as greater level of edema and the presence of hematoma in the group with a complete closure. Danda *et al*. (7) made a split-mouth study and concluded that the secondary closure of the wound produces less postoperative pain and swelling than the group with a primary closure. By contrast, Bello *et al*. (5) reported lower swelling in the group with a partial closure of the wound but they did not find differences regarding trismus or pain.

Other authors (3,9,10) have evaluated the secondary closure of the wound without sutures obtaining slightly different results. Waite and Cherala (3) studied the outcome from not suturing a small “V” shaped flap in 1280 extracted molars from 366 patients and obtained satisfactory results in terms of postoperative complications. Conversely, Osunde *et al*. (10) performed a study comparing the effect from suturing with not suturing and they found a reduction in the severity of pain at the first and second days in the group with no sutures, although at the seventh day the results were equal to the suture group. They did no report differences regarding postoperative swelling and trismus between groups. Contrary to the last, a similar study published by Hashemi *et al*. (9) reported lower scores of pain and swelling in the group without suture. The benefits from a no suture technique are the lower cost, less operative time, less manipulation of soft tissue and hence, less postoperative morbidity (3,10). Distinct authors (9,10) suggest that the creation of a drainage pathway for inflammatory exudate helps to reduce symptoms and postoperative complications. Total wound closure can act as a one-way valve that permits food debris to enter the socket but does not allow it to escape. This predisposes to local infection, inflammation, edema and pain (3,5,16). The main drawback of suture-less is that healing may be delayed. In addition, there may be high potential for the formation of a periodontal pocket in relation to the adjacent second molar (10). However, a recent metaanalysis (4) concludes that there are no significant differences on the outcome between complete and partial wound closure and it also refers that the available studies are heterogeneous and do not produce high level of scientific evidence.

With the aim of controlling the immediate effects and to prevent complications after the impacted lower third molar removal, there are other methods described as the excision of a wedge of distal mucosa of the second molar (15,20), that seems to reduce postoperative morbidity, and the use of a method of drainage (2,13,17-19), although some controversy exists regarding the effect in postoperative variables after third molar surgery.

The flap design seems to be a factor that can also affects the postoperative course. Some studies (8,11) compared the use of an envelope flap against a triangular one and they did not demonstrate significant differences in postoperative variables. However, a study made by Baqain *et al*. (6) obtained better results regarding trismus and swelling with the use of an envelope flap. Likewise, a comparative study performed by Sanchis-Bielsa *et al*. (16) proved that the postoperative course was worse when using a reflection flap for healing by first intention than only approximating the borders of the wound.

The results of this study show that there are no statistically significant differences in terms of pain, swelling and trismus between the complete and partial closure in which the usual suture technique is simplified and no complete suture of the relieving incision is performed. However, these variables are less significant with the partial closure of wound, reducing the surgery length.
